# Volume of Surgical Freedom: The Most Applicable Anatomical Measurement for Surgical Assessment and 3-Dimensional Modeling

**DOI:** 10.3389/fbioe.2021.628797

**Published:** 2021-04-13

**Authors:** Lena Mary Houlihan, David Naughton, Mark C. Preul

**Affiliations:** The Loyal and Edith Davis Neurosurgical Research Laboratory, Department of Neurosurgery, Barrow Neurological Institute, St. Joseph’s Hospital and Medical Center, Phoenix, AZ, United States

**Keywords:** neuroanatomical quantitation, surgical target structure, surgical access corridor, volume of surgical freedom, 3D modeling

## Abstract

Surgical freedom is the most important metric at the disposal of the surgeon. The volume of surgical freedom (VSF) is a new methodology that produces an optimal qualitative and quantitative representation of an access corridor and provides the surgeon with an anatomical, spatially accurate, and clinically applicable metric. In this study, illustrative dissection examples were completed using two of the most common surgical approaches, the pterional craniotomy and the supraorbital craniotomy. The VSF methodology models the surgical corridor as a cone with an irregular base. The measurement data are fitted to the cone model, and from these fitted data, the volume of the cone is calculated as a volumetric measurement of the surgical corridor. A normalized VSF compensates for inaccurate measurements that may occur as a result of dependence on probe length during data acquisition and provides a fixed reference metric that is applicable across studies. The VSF compensates for multiple inaccuracies in the practical and mathematical methods currently used for quantitative assessment, thereby enabling the production of 3-dimensional models of the surgical corridor. The VSF is therefore an improved standard for assessment of surgical freedom.

## Introduction

### Importance of Quantitative Anatomy

Anatomy is the foundation of medical understanding. Medical practice has evolved through the continual scrutiny of biological structure and physiologic function (Acar et al., 2005; Elhadi et al., 2012; Arraez-Aybar et al., 2015). As the merits of anatomical scrutiny in disease therapy were elucidated, the drive to be able to discriminate between “normal” and “abnormal” biological arrangement increased. This development resulted in the advent of quantitative anatomical research, the objective of which was to measure the complexity of human architecture.

Biological variability is an accepted reality (Kreutz and Timmer, 2009; Higdon, 2013) and a key aspect of managing pathologic processes. The aim of quantifying anatomy has been to identify the most reproducible homogenous model of specific organ systems, thereby establishing principles in biological structure and physiology. The establishment of these principles allowed for the appreciation of abnormal morphology and pathologic processes. The criteria for what now constitutes the so-called normal anatomy has been used in every aspect of medical education, investigation, translational research, and treatment development (Iaizzo et al., 2013).

Anatomical competency is of the utmost importance in surgical practice (Aziz and Mansor, 2006; Burgess and Ramsey-Stewart, 2015). It is the cardinal infrastructure upon which the knowledge base for all surgeons is founded and subsequently evolves (Selcuk et al., 2019). The surgeon must be aware of standardized structures and their spatial positioning, associated variations, and physiologic sequelae. The efforts and discoveries of anatomists have spurred pivotal breakthroughs in surgical and medical treatment (Melly et al., 2018; Iorio-Morin and Mathieu, 2020) as well as in the development of basic scientific progression and understanding of the disease process (Barth and Ray, 2019).

### Quantitation of Surgical Feasibility

Quantitative anatomy is the method the surgeon uses to assess the surgical benefits and disadvantages of different surgical approaches. Studying quantitative anatomy improves the techniques of neurosurgery and other related surgery disciplines. This process allows the surgeon and related personnel to assess, plan, and select the optimal intervention or surgical approach specific to the pathology, thereby improving surgical outcomes for patients. Neuroanatomy is especially relevant and critical because the structural, functional, and physiologic components are often small in dimensional relation and are particularly intertwined. There is little room for error in neurosurgery; all system components represent a significant function, usually reflected in their structural integrity. The intricacy of preserving structural eloquence in the nervous system is further echoed in the surgical parameters the neurosurgeon must use. Dr. Albert Rhoton Jr. revolutionized the field of neuroanatomy, making neurosurgery “more accurate, gentle and safe” (Matsushima et al., 2018) not only by establishing key concepts in microsurgical anatomy but also by extrapolating the findings to a surgical approach-specific setting. This innovation enhanced the relevance of anatomy in surgical planning and led to the development of integral concepts that surgeons now use to determine the efficacy of the surgical approach.

The ability to manipulate surgical instruments is an important criterion in comparing surgical approaches and selecting the optimal one. Freedom of movement is especially relevant in neurosurgery, where surgical access through the cranium and into the deep areas of the brain is often restricted. When accessing the most extreme limitations of a surgical corridor, the neurosurgeon encounters parenchymal, bony, musculocutaneous, and neurovascular structures that define the boundaries. The degree of manipulation within these parameters is specific to the approach and delicacy of the structure, the appreciation of which is only possible with extensive knowledge of the circumferential anatomy. An appreciation of these anatomical confines is second nature for the trained neurosurgeon; nonetheless, the mapping of surgical corridors specific to these structures has not yet been robustly completed.

When neurosurgery is performed using an operating microscope for magnification, the movement of surgical instruments to work on pathoanatomical structures may be in increments of millimeters. Small surgical corridors, microscopic anatomy, surgical depth, and impaired visualization all impose limitations on neurosurgical interventions. These are the principal surgical criteria that influence the neurosurgical decision-making process. Technological advances have broadened the available visualization options, with the microscope, endoscope, and exoscope all possessing specific benefits and disadvantages. This additional component must be assimilated into the surgical decision-making process (Jane, 2013; Belykh et al., 2018; Herlan et al., 2019). Only through quantitative anatomical assessment specific to these surgical parameters can neurosurgeons increase their insight and proficiency in neurosurgical techniques and operative interventions.

Analysis of the surgical corridor is critical to assessing the validity of any surgical approach. From a neurosurgical perspective, the ideal corridor to the structure of interest should be minimally invasive, with minimal morbidity and mortality, and it should be cosmetically satisfactory (Castelnuovo et al., 2013). Conceptually, the best surgical corridor combines the maximal room for instrument maneuverability and maximal visualization with the shortest distance to the target of interest. Instrument maneuverability and visualization are primary concerns; thus, a means is required for quantitatively assessing the spatial and morphometric advantages of the surgical corridor, in addition to considering the distance to the STS. This metric enables consideration of the influence of neuroanatomical structures on the feasibility of the corridor, as well as the ability of the neurosurgeon to function and complete the specific intervention. How well the neurosurgeon can operate and manipulate instruments with respect to the surgical approach directly influences the patient’s outcome.

The measurement by which instrument maneuverability is quantified is termed “surgical freedom.” The first description of surgical freedom was noted in 2000 (Horgan et al., 2000; Spektor et al., 2000). Stereotactic data gathered using a frameless stereotactic navigation device was used to produce a quantitative measurement of the area available for instrument maneuverability. The 3D coordinate data of the region were used to calculate the area by the summation of triangular areas. Twenty years later, this method remains the crux of neuroanatomical quantitation and a key determinant of the feasibility of any surgical approach (Pillai et al., 2009; Elhadi et al., 2014, 2015).

### Surgical Freedom

Surgical freedom is defined in the medical literature as the maximum allowable working area at the proximal end of a probe with the distal end on the target structure (Horgan et al., 2000; Spektor et al., 2000; Noiphithak et al., 2018). The goal of this procedure is to assess the maneuverability of an instrument and provide the operator with insight into how realistic and appropriate it is to use a specific access corridor while also allowing for the comparison of surgical approaches.

**FIGURE 1:**
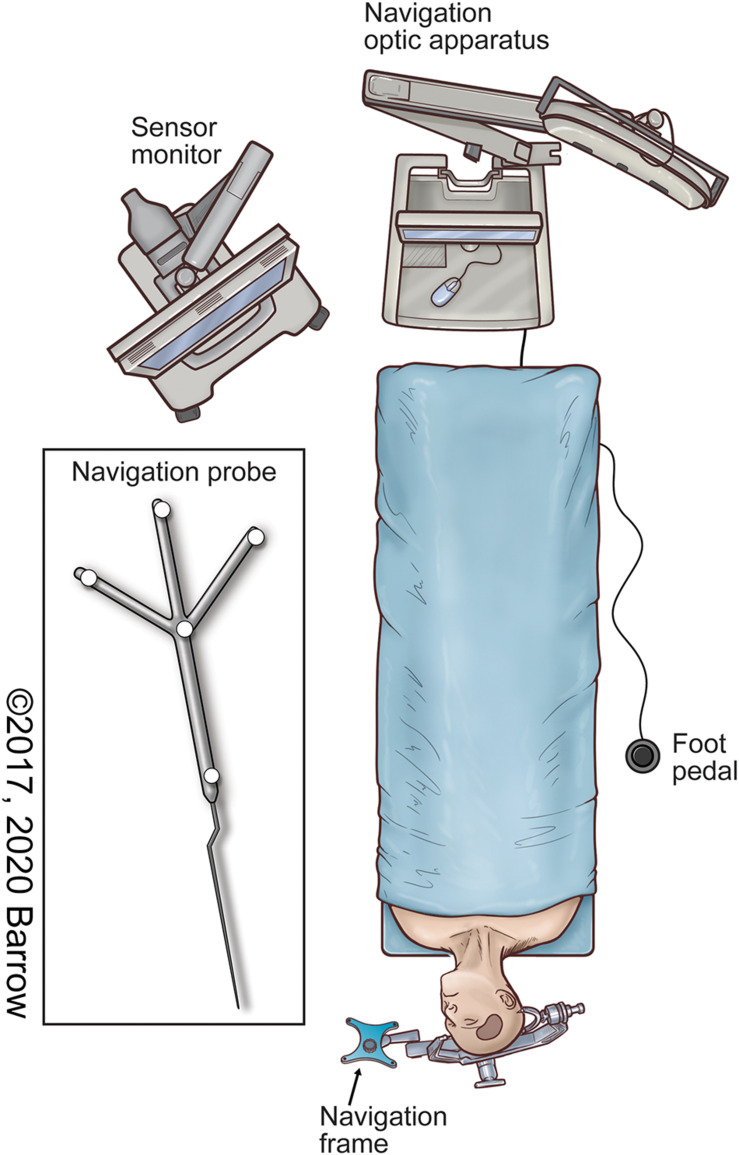
Illustration of the neuronavigation system in the surgical setup commonly used to localize both pathologic and anatomically pertinent structures using stereotaxis. Used with permission from Barrow Neurological Institute, Phoenix, Arizona.

Neuroanatomists and neurosurgeons use specific methods to measure surgical freedom:

• The cadaveric head specimen is fixed in a rigid head holder.
• A stereotactic navigation system (Figure 1) is used to acquire the 3D coordinates of the target points for each surgical approach being analyzed, specific to the intracranial structure or region of interest.

• The distal end of the surgical instrument is placed on the target structure.
• For a quadrangular area measurement, the surgical instrument is moved as far mediocranially, mediocaudally, laterocranially, and laterocaudally as possible for four or more points to represent the most extreme limits of the surgical corridor specific to the approach. At all times, the distal end of the probe is not moved from the target structure.
• The coordinates of the surgical instrument’s proximal tip are obtained with the navigation probe when the tip of the instrument is at the most extreme position.
• The area bounded by the coordinate data points from the probe’s proximal tip at the extreme limits is calculated by dividing the bounded area into triangles in which the data points form the triangle vertices, then calculating the sum of the areas of all the triangles using Heron’s formula (Weisstein).
• The result is represented in either square millimeters or square centimeters.

This method produces an area that is used as a metric for surgical freedom. The standard process is to collect four, but occasionally six, data points. The distribution of these points, as dictated by the measuring researcher, is usually along the extrema of the surgical corridor, which does not necessarily take into account any structural components, or the lack thereof, between the points. Previous methods have tended to impose regular, symmetrical shapes on the measurement data to simplify the shape of the access corridor. In reality, however, the borders of any surgical corridor are never symmetrical, and they are never a perfect shape that can be represented by conventional shapes.

This method aims to quantitatively portray the range of motion of an instrument during a surgical intervention to illustrate the feasibility and functionality of the surgical approach and the surgical corridor specific to the target structure. However, multiple inaccuracies are associated with this calculation method from a practical, mathematical, and application perspective: (1) The measured data points are not coplanar, which distorts the perceived area of surgical freedom. (2) The surgical freedom metric is dependent on the length of the probe that is used to capture data. This variation across the literature precludes interstudy comparisons, which weakens the scientific robustness of such studies and impairs reproducibility. (3) Measurement inaccuracy can result in substantial variation in the measured area. (4) Surgical corridors are irregular and cannot be fully expressed using simple shapes. (5) A 2-dimensional (2D) shape does not allow for visualization of a 3D surgical corridor.

In terms of visualization, the area of surgical freedom is not an optimal representational concept of the surgical approach corridor because the area is a 2D measurement, and neurosurgical approaches and corridors are volumetric, or 3D, shapes. Surgical freedom is arguably the most important technical parameter dictating the surgical approach and selection process specific to an anatomical target. Quantitatively analyzing surgical freedom allows the neurosurgeon to proceed in a more informed fashion by comparing the numerical values with those of different approaches to the same anatomical target. What is not taken into consideration by this method is the fact that surgical instruments are not deployed in a 2D area but rather in an irregularly shaped 3D corridor.

Due to the nature of the surgical site, the surgical corridor transitions from a region of large maneuverability at the surgical entry point to an apex of minimal freedom at the target structure. At present, an instrument’s maneuverability within the surgical corridor is estimated by the angle formed at the apex of the corridor (the angle of attack) in one or two planes, usually vertical or horizontal. The angle of attack is another anatomical metric neurosurgeons use to evaluate an instrument’s maximal working ability in one or more planes where the instrument will be most frequently deployed. This information gives specific insight regarding the instrument’s operational freedom, which may not be evident when assessing the numerical value produced by the present method of calculating surgical freedom. However, this method produces a limited representation of the 3D shape of the surgical corridor.

The deficits in estimating this surgical principle are exemplified by the illustrations published in the neurosurgery literature. These illustrations broadly represent the surgical corridors, denoting general shapes and trajectories garnered from the neurosurgeon’s experience, but they lack anatomical, spatial, and surgical accuracy. Surgical freedom should be defined by the whole expanse of the surgical corridor and should not be limited to its 2D infrastructure. For all these reasons, we have endeavored to improve upon the imprecision in currently accepted methodology for this crucial method of quantitative surgical anatomy.

### Volume of Surgical Freedom

The volume of surgical freedom (VSF) is defined as the maximal available working volume with respect to a specific surgical corridor and target structure. VSF is a new methodology that produces the optimal qualitative and quantitative representation of an access corridor and provides the neurosurgeon with an anatomical, spatially accurate, and clinically applicable metric. From this representation, 3D visualization of the surgical corridor is possible.

The VSF metric uses a normalized calculation to reduce error and allow for direct comparison among measurements. This calculation is achieved by measuring the volume of the irregular-based cone model of the surgical corridor, with the irregular base of the cone at a fixed distance from the apex. This report details a novel approach for surgical anatomy quantitation, the anatomical experiment used to investigate its validity, and the key steps in producing a mathematically and spatially superior model of the approach corridor.

## Materials and Equipment

### Anatomical Specimen Preparation

Cranial dissections of 14 cadaveric specimens were completed to investigate the data-collection process and for logistical and surgical representation. The cadaveric heads were fixed with a customized alcohol-based solution as a preservative. Colored silicone was incrementally injected into the cerebral vasculature, with the arteries represented by red and the veins represented by blue. This differentiation allowed for clearer interrogation of the intracranial structures and surgical target structure (STS). Each head was rigidly fixed in a head holder while measurements were obtained.

**FIGURE 2:**
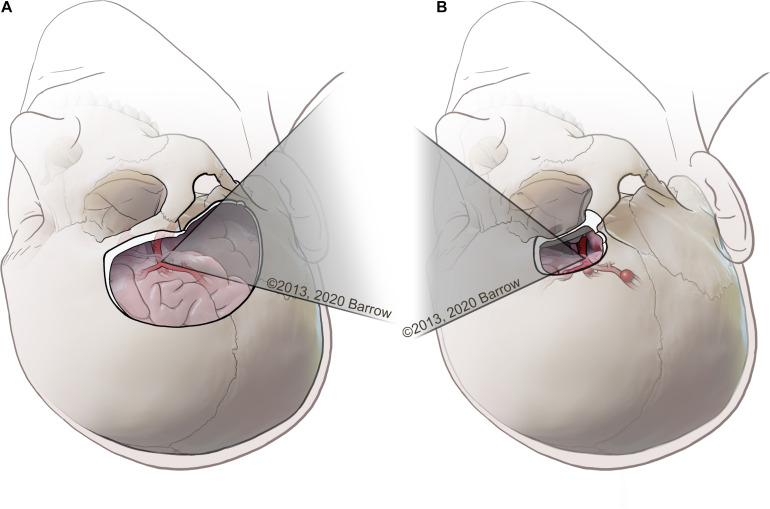
**(A)** Example of a pterional transcranial approach, in which the surgeon uses a lateral trajectory that usually traverses the natural fissures of the brain to access complex paramedian regions. **(B)** Example of a supraorbital approach, with an incision made along the eyebrow approaching the skull base from a more anterolateral perspective, tracking medially along the bone of the anterior cranial fossa that represents the roof of the orbit. Used with permission from Barrow Neurological Institute, Phoenix, Arizona.

Dissections were completed by the first author, a neurosurgery resident competent in the two selected approaches. Dissection was completed using a clinical-grade neurosurgical operating microscope (Zeiss OPMI Pentero, Carl Zeiss Meditec AG, Oberkochen, Germany). The two open transcranial neurosurgical approaches selected to model this quantitative methodology were the standard pterional craniotomy and the supraorbital craniotomy (Figure 2). These approaches are two of the most common neurosurgical corridors used to access deep paramedian structures and regions of surgical complexity. These anatomical areas are of particular interest in the context of anatomical quantitation because of the need to avoid injury to critical neurovascular structures.

### Neuronavigation System

A neuronavigation system (StealthStation S7 Surgical Navigation System; Medtronic, Dublin, Ireland) was used to acquire predetermined data points. Neuronavigation uses the principle of stereotaxis. The neuronavigation system uses Cartesian coordinates to divide the geometric volume of the brain into three imaginary intersecting spatial planes (axial, sagittal, and coronal) that are orthogonal to each other. Any point within the brain can be specified by measuring its distance along these three intersecting planes. Neuronavigation uses the reference of this coordinate system in parallel with 3D images of the brain displayed on the console of the computer workstation to provide guidance to the corresponding anatomical locations using medical images (Bas̨arslan and Cüneyt, 2014).

Institutional review board approval was not required for this cadaveric laboratory investigation.

### Methodology Calculator and 3D Modeling Software

The methodology described herein was implemented as a calculation tool in Excel for Office 365 (Microsoft, Redmond, WA, United States). An Excel spreadsheet was used to perform all of the calculation steps described in this paper, apart from the calculation of the best-fit plane. The generalized reduced gradient nonlinear engine of Microsoft Excel Solver was used to calculate the least-squares best-fit plane for the data points. The Excel spreadsheet calculation tool was used to calculate the normalized volume of the surgical corridor (normalized VSF) using the measurement data as an input. In addition to calculating the VSF metric using Excel, the VSF data were modeled using a student license for the 3D modeling software Solidworks 2020 (Dassault Systèmes, Vélizy-Villacoublay, France). The modeling software was used to create 3D renderings of the surgical corridors from the measurement data to visualize the surgical corridor for each dataset. The 3D models were also superimposed onto microscope images of anatomical approaches to illustrate the surgical corridor to the structure of interest.

## Methods

### Data Collection

**FIGURE 3:**
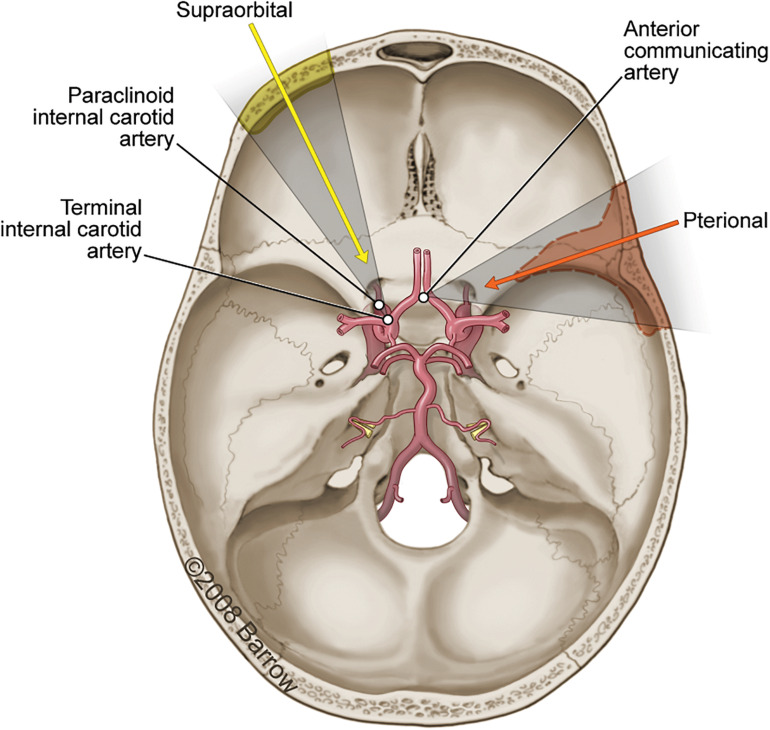
Axial view illustration of the skull base showing the primary vasculature supplying the brain. The three surgical structures of interest accessible using either a pterional craniotomy (*orange arrow and shading*; *dashed line*) or supraorbital craniotomy (*yellow arrow and shading; solid line*) are the paraclinoid internal carotid artery (ICA), the terminal ICA, and the anterior communicating artery. Used with permission from Barrow Neurological Institute, Phoenix, Arizona.

A pterional craniotomy was conducted on seven cadaveric specimens, and a supraorbital craniotomy was conducted on seven cadaveric specimens. Predetermined STSs were selected by the first author. To illustrate the methodology, three surgical targets (Figure 3) were identified that are common to both approaches: (1) paraclinoid internal carotid artery (ICA), (2) terminal ICA, and (3) anterior communicating artery (ACoA) complex.

**FIGURE 4:**
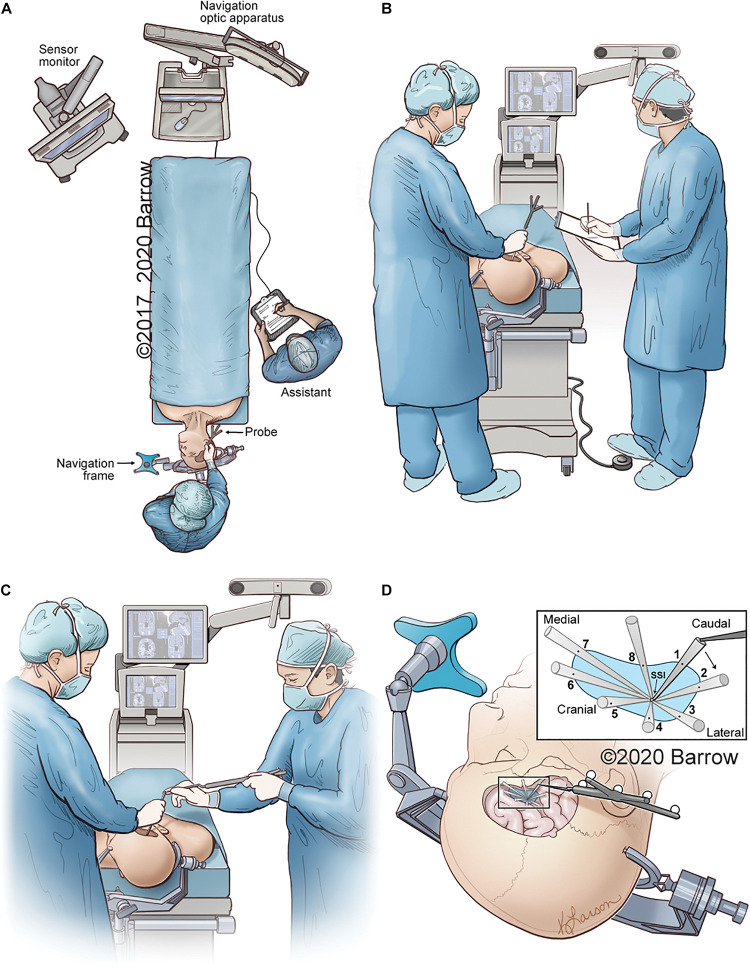
Illustrative depiction of the data collection process. **(A)** Position of the neuronavigation system, the cadaver, the neurosurgeon with probe, and the assistant recording data. **(B)** The neurosurgeon obtains coordinates of the STS with the tip of the neuronavigation probe on the STS. The coordinates, as depicted on the monitor, are recorded by the assistant. **(C)** While the surgeon holds an instrument with its distal end on the STS, the assistant holds the neuronavigation probe and places its distal tip on the proximal end of the surgeon’s instrument to obtain the 3D coordinates of the instrument’s proximal end in space. **(D)** Eight data points are sequentially collected that represent the maximal allowable parameters of the surgical corridor. The data points are obtained by placing the tip of the navigation probe on the proximal end of the surgical instrument in the position marking a specific boundary point. Points 1 and 5 represent the craniocaudal maximal angle of attack, whereas points 3 and 7 can be used to represent the mediolateral angle of attack. STS, surgical target structure. Used with permission from Barrow Neurological Institute, Phoenix, Arizona.

The data points required to calculate the VSF and produce a spatially and anatomically accurate model were collected for each STS and both the pterional and supraorbital surgical approaches. Figure 4 depicts the collection process for all data points.

Interrater and intrarater variability were accounted for by recording the STS VSF for each specimen and approach a minimum of three times by multiple qualified neurosurgery residents. This replication ensured reproducibility of the method, as well as a larger pool of measurements to assess our methodology’s advantages and limitations. VSF results are reported as cubic millimeters, and each result was normalized to a height of 10 mm from the STS.

### Mathematical Methodology

The VSF was calculated by modeling the surgical corridor as a cone with an irregular base. The STS is the apex of the cone, and the points measured at the extrema of the maneuverability of the instrument compose the base of the cone. For this study, we measured eight extrema points around the base of the cone, but the calculation methodology is equally applicable to more or less than 8 points. The 3D coordinate data from the measurements were used to calculate the volume of the irregular-based cone. This method can be summarized as follows:

(1) Calculate a best-fit plane to the extrema data points, which best represents the plane of the base of the cone.
(2) Translate the best-fit plane in 3D space to a fixed perpendicular distance from the apex point (normalized height), maintaining the slope of the plane.
(3) Translate the extrema data points onto the best-fit plane, along the line between the measured point and the apex point.
(4) Convert the 3D coordinates of the data points to a 2D coordinate system on the best-fit plane.
(5) Use the 2D coordinates to calculate the area of the irregular polygon enclosed by the data points.
(6) Calculate the perpendicular height from the best-fit plane to the apex point.
(7) Calculate the volume of the irregular-based cone from the area and the perpendicular height.

**FIGURE 5:**
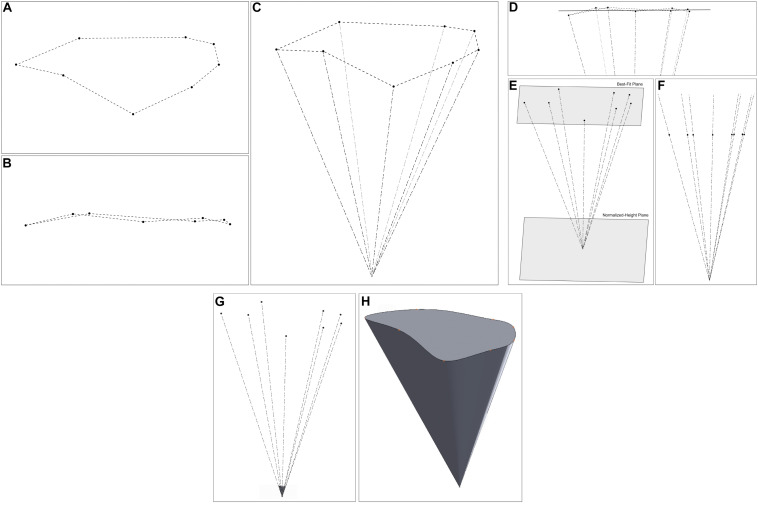
3D modeling of the steps of the volume of surgical freedom methodology. **(A)** A plan view of the data points in 3D space. **(B)** An elevation view of the points, illustrating that they are not on a single plane. **(C)** A view of the data points and apex point in space. **(D)** The best plane fitted to the data points. **(E)** A view of the normalized-height plane, at a fixed distance from the apex. **(F)** An elevation view of the data points after translation to the normalized-height plane. **(G,H)** The 3D representation of the shape of the surgical corridor **(G)** relative to the measured data points **(H)** standalone view of the corridor model. Used with permission from Barrow Neurological Institute, Phoenix, Arizona.

This methodology calculates a normalized volume by calculating the volume of the cone with an irregular base, modeling the surgical corridor at a fixed height from the apex point (Figure 5). This methodology was conceived to reduce the effects of measurement inaccuracy and measurement probe length on the calculated VSF value.

#### Calculation of Best-Fit Plane

The best-fit plane was fitted to the data points using the least-squares method. The best-fit plane was considered to be the plane that results in the minimum sum of the squares of the perpendicular distances between each data point and the plane.

The problem is nonlinear, so a nonlinear solver was used to optimize the best-fit plane. An average plane was first calculated to use as an initial condition in the solver. The plane intersecting each subset of three points was calculated using various combinations of three data points from the set of extrema data points. The average plane equation coefficients of all the calculated planes were used as the coefficients of the average for the initial condition in the solver.

#### Calculating a Plane From Three Points

The general equation of a plane can be expressed as follows:

A⁢x+B⁢y+C⁢z+D=0,

where *A, B*, and *C* are the coefficients of the slope in the directions of the *x, y*, and *z* axes, respectively, and *D* is the coefficient representing the distance from the plane to the origin.

Calculating a plane from three points *P*_1_ = (*x*_1_,*y*_1_,*z*_1_), *P*_2_ = (*x*_2_,*y*_2_,*z*_2_), and *P*_3_ = (*x*_3_,*y*_3_,*z*_3_) first requires calculating the two vectors between the three points:

$$\vec{P_{1}\,P_{2}}=P_{2}-P_{1},$$

$$\vec{P_{1}\,P_{3}}=P_{3}-P_{1}.$$

The *A, B*, and *C* coefficients of the plane can then be calculated from the cross-product of the two vectors:

$$\left[A\,B\,C\right]=\vec{P_{1}\,P_{2}}\times \vec{P_{1}\,P_{3}}.$$

The coefficient *D* can then be calculated from the plane equation by using one of the points (*P*_1_):

$$D=-\left(A\,x_{1}+B\,y_{1}+C\,z_{1}\right)$$

#### Calculating the Average Plane

After the plane equation of *n* planes has been calculated, the average plane can be calculated from the average coefficients:

$$A_{\text{average}}\,x+B_{\text{average}}\,y+C_{\text{average}}\,z+D_{\text{average}}=0,$$

where

$$A_{\text{average}}=\frac{A_{1}+A_{2}+\ldots +A_{n}}{n},$$

$$B_{\text{average}}=\frac{B_{1}+B_{2}+\ldots +B_{n}}{56},$$

$$C_{\text{average}}=\frac{C_{1}+C_{2}+\ldots +C_{n}}{n},\text{and}$$

$$D_{\text{average}}=\frac{D_{1}+D_{2}+\ldots +D_{n}}{n}.$$

#### Running the Excel Solver to Calculate the Least-Squares Best-Fit Plane

Because the least-squares calculation of the best-fit plane is a nonlinear problem, a nonlinear solver was used to calculate the coefficients of the least-squares fitting plane. The coefficients *A*_average_, *B*_average_, *C*_average_, and *D*_average_ were used as the initial conditions for the solver. The distance *d* between each point *P* = (*x*,*y*,*z*) and the plane *Ax* + *By* + *Cz* + *D* = 0 is obtained by

$$d=\frac{\left|A\,x+B\,y+C\,z+D\right|}{\sqrt{A^{2}+B^{2}+C^{2}}}.$$

The nonlinear solver then solved for the coefficients *A, B, C*, and *D*, which minimized the expression

$$\sum_{p=1}^{n}{\left(\frac{A\,x_{p}+B\,y_{p}+C\,z_{p}+D}{\sqrt{A^{2}+B^{2}+C^{2}}}\right)}^{2},$$

where *n* is the number of data points to which the plane is being fitted.

#### Normalizing the Best-Fit Plane

Calculating the cone’s cross-sectional area at a fixed distance from the apex required a new normalized-height plane, which is parallel to the cone’s base, at the required distance from the apex point. After the best-fit plane *Ax* + *By* + *Cz* + *D* = 0 is calculated using the nonlinear solver, this plane is translated into 3D space so that the perpendicular distance between the apex point and the plane is the required normalized height of the cone.

Because the plane is parallel to the base of the cone (i.e., the calculated best-fit plane for the data points), the *A, B*, and *C* plane coefficients will be the same for both planes. Given this constraint, the formula for the perpendicular distance between the normalized-height plane *Ax* + *By* + *Cz* + *D*_norm_ = 0 and the apex point *P*_*a*_ = (*x*_*a*_,*y*_*a*_,*z*_*a*_) can be rearranged to give the following:

$$D_{\text{norm}}=h_{\text{norm}}\,\left(\sqrt{A^{2}+B^{2}+C^{2}}\right)-\left(A\,x_{a}+B\,y_{a}+C\,z_{a}\right).$$

The least-squares best-fit plane was used as the plane for the base of the cone model.

#### Translating the Points to the Best-Fit Plane

After the normalized-height plane is calculated, the points must be translated onto this plane to calculate the area of the base of the cone model. Maintaining the shape of the cone requires the points to be translated onto the normalized-height plane along the line between each point and the apex. This method ensures that the cross-section profile of the shape of the cone is unaltered when the points are translated.

The vector between each data point *P* = (*x*,*y*,*z*) and the apex point *P*_*a*_ = (*x*_*a*_,*y*_*a*_,*z*_*a*_)is given by the following:

$$\vec{P\,P_{a}}=P_{a}-P.$$

A multiplication factor, *t*, representing the distance between the point and the best-fit plane *Ax* + *By* + *Cz* + *D*_norm_ = 0is calculated as follows:

$$t=\frac{-\left(A\,x_{a}+B\,y_{a}+C\,z_{a}+D_{\text{norm}}\right)}{A\,x_{0}+B\,y_{0}+C\,z_{0}}.$$

The coordinates of the point after translation onto the best-fit plane are then calculated by

$$P^{\prime }=\left[x^{\prime }\,y^{\prime }\,z^{\prime }\right]=\left[t\,x\,t\,y\,t\,z\right].$$

The translation was performed for all *n* data points to obtain the translated points $P_{1}^{\prime },P_{2}^{\prime }\,\ldots \,P_{n}^{\prime }$.

#### Calculating the Area Enclosed by the Translated Points

The shoelace formula (Weisstein) was used to calculate the area of the base of the cone model. This formula calculates the enclosed area of an irregular polygon from the 2D coordinates of the vertices of the polygon. Calculating the area using this method required mapping the translated 3D points to a 2D coordinate system on the normalized-height plane.

The choice of the origin was arbitrary, so the 2D plots of the data points were simplified by taking the centroid of the data points as the new origin. The centroid is calculated by taking average *x, y*, and *z* coordinates of all data points as follows:

$$\text{Centroid}\,O=\left[x_{O}\;y_{O}\;z_{O}\right]=\left[\frac{x_{1}+x_{2}+\ldots +x_{n}}{n}\,\frac{y_{1}+y_{2}+\ldots +y_{n}}{n}\,\frac{z_{1}+z_{2}+\ldots +z_{n}}{n}\right].$$

Two perpendicular axes on the plane were created by defining two vectors on the normalized-height plane using the centroid *O*, and two of the translated points $P_{1}^{\prime }$ and $P_{2}^{\prime }$:

$$\vec{v}=P_{1}^{\prime }-O,$$

$$\vec{u}=P_{2}^{\prime }-O.$$

A vector $\vec{w}$ is calculated that is perpendicular to both $\vec{u}$ and $\vec{v}$:

$$\vec{w}=\vec{u}=\vec{v}.$$

A new vector, $\vec{u^{\prime }}$, is then calculated perpendicular to both $\vec{v}$ and $\vec{w}$:

$$\vec{u^{\prime }}=\vec{v}=\vec{w}.$$

The unit vectors $\hat{v}$ and $\hat{u^{\prime }}$ are then calculated:

$$\hat{v}=\frac{v}{\left|v\right|},\hat{u^{\prime }}=\frac{u^{\prime }}{\left|u^{\prime }\right|}.$$

This series of calculations results in two perpendicular vectors on the normalized-height plane that are then used as the axes for the 2D coordinate system. Each 3D data point *P*′ is converted to a 2D point *P*_*2D*_ by calculating the dot product of each axis vector with the vector between the origin and the point $\vec{O\,P^{\prime }}$:

$$P_{2\,D}=\left(x_{2\,D}y_{2\,D}\right)=\left(\hat{u^{\prime }}.\vec{O\,P^{\prime }}\hat{v}.\vec{O\,P^{\prime }}\right).$$

With the data points mapped to a 2D coordinate system, the area of the shape enclosed by the points is calculated using the shoestring formula:

$$A=\frac{1}{2}\left(|x_{1}x_{2}y_{1}y_{2}|+|x_{2}x_{3}y_{2}y_{3}|++|x_{n}x_{1}y_{n}y_{1}|\right),$$

where |*x*_*n*_*x*_*n* + 1_*y*_*n*_*y*_*n* + 1_| is the determinant of the matrix, given by

$$\left|x_{n}\,x_{n+1}\,y_{n}\,y_{n+1}\right|=x_{n}\,y_{n+1}-y_{n}\,x_{n+1}.$$

#### Calculating the Perpendicular Height of the Cone Shape

The perpendicular height of the cone shape, *h*, is simply the perpendicular distance between the best-fit plane *Ax* + *By* + *Cz* + *D* = 0 and the apex point*P_a_* = (*x*_*a*_,*y*_*a*_,*z*_*a*_):

$$h=\frac{\left|A\,x_{a}+B\,y_{a}+C\,z_{a}+D\right|}{\sqrt{A^{2}+B^{2}+C^{2}}}.$$

#### Calculating the Volume of the Cone Shape

The volume of the cone shape can be calculated from the area enclosed by the points on the best-fit plane (*A*) and the perpendicular height of the cone shape (*h*):

$$\text{Volume}=\frac{1}{3}=A=h.$$

This volume is reported as cubic millimeters.

#### Normalized Volume of Surgical Freedom

The volume calculation depends on the length of the probe used to obtain the point data because the length of the probe determines the height of the cone shape. If this factor is removed from the calculation, then the resulting VSF values will be directly comparable to all other calculations using this spreadsheet or methodology, regardless of the length of the probe used to take the measurements.

### Modeling Methodology

The 3D models of the surgical corridors were generated from the coordinates of the extrema points after translation onto the normalized-height plane and from the coordinates of the apex point. Although the mathematical calculation of the area assumes straight lines between each of the extrema data points when calculating the enclosed area of the base of the cone, the 3D model of the surgical corridor used curved splines between each of the extrema points to better visualize the shape of the surgical corridor observed in the specimen.

### Measurement Inaccuracy Analysis

Because the original measurement data points were not coplanar, we investigated their effect on the calculation of the area bounded by the points. For an illustrative data set, the area bounded by the measured data points was calculated using Heron’s formula. The data points were then translated onto the best-fit plane, and the area bounded by these translated points was calculated using the same method.

As of this writing, 174 individual VSF measurements have been completed in the neurosurgical research laboratory to explore multiple neurosurgical approaches. A 190-mm probe was used for all measurements. For each set of measurement data, the average probe length was calculated by averaging the calculated distance from each of the eight coordinates to the apex coordinate. The average minimum and maximum probe lengths with standard deviations (SDs) were identified for the 174 measurement samples.

Analysis of the effect of the probe length on the calculated volume of the surgical corridor required the calculation of the volume of a cone from the best-fit plane of the measurement data points to the apex point. The cone shape was maintained while the cone height was increased by 5 mm, and the data points were translated onto the plane 5 mm farther away from the apex. This translation created a new data set representing the same surgical corridor as that measured by a longer probe. The volume of the cone was then calculated again from the best-fit plane to the new data set representing a longer probe. Data analysis was completed in Microsoft Excel.

## Results

### Quantitation and Modeling

This methodology generated two useful products: a mathematically robust quantitation of the surgical freedom of a neurosurgery instrument and a 3D spatially accurate model of the surgical corridor that takes into account irregular neuroanatomical parameters. This process gives direct quantitative information to allow for the comparison of surgical approaches. VSF is expressed in cubic millimeters. By default, the VSF also produces the craniocaudal and mediolateral angles of attack. The spatially accurate model obtained with the VSF provides a visual representation of this information, elucidating the breadth of maneuverability specific to all planes.

Table 1 depicts the comparative quantitative results of a set of measurements for specific anatomical STSs and pterional and supraorbital approaches. These illustrative examples show that the pterional craniotomy provides a larger VSF for all three STSs than the VSF provided by the supraorbital craniotomy. Thus, if any of these structures must be accessed, the pterional craniotomy would be the superior corridor because it provides an increased VSF and an increased angle of attack in both the craniocaudal and mediolateral dimensions.

**TABLE 1:** Comparative volumetric results of the VSF for measuring instrument maneuverability specific to the surgical target structure and approach*.

Surgical target structure	VSF, mm^3^ NU		Craniocaudal Angle of Attack, degrees		Mediolateral Angle of Attack, degrees	
			
	Pterional	Supraorbital	Pterional	Supraorbital	Pterional	Supraorbital
Paraclinoid ICA	165.88	43.83	36.72	20.86	50.55	37.40
Terminal ICA	50.69	31.01	22.63	16.29	27.59	31.62
ACoA complex	38.34	15.66	12.64	14.50	31.69	17.44

*ACoA, anterior communicating artery; ICA, internal carotid artery; NU, normalized unit; VSF, volume of surgical freedom.* *Craniocaudal and mediolateral angles of attack were also produced using this measurement system.

**FIGURE 6:**
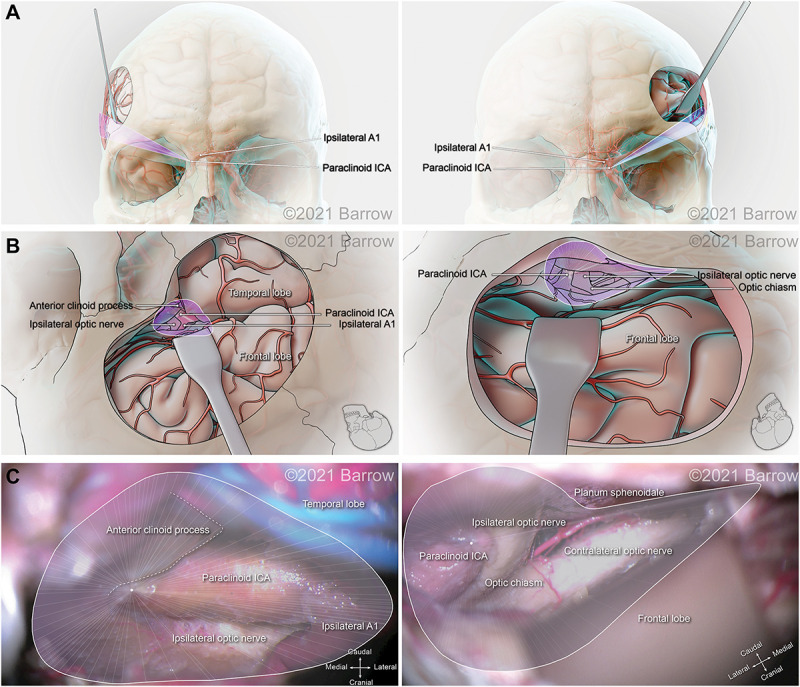
Illustration depicting 3D modeling of the surgical corridor to the paraclinoid internal carotid artery (ICA) from a pterional approach (left) and a supraorbital approach (right). **(A)** The anterior of the surgical corridor. **(B)** The surgical anatomy as visualized specific to the surgical corridor model. **(C)** The surgical view of the cadaveric anatomy, which is in continuity with the surgical corridor model parameters (pterional: VSF = 165.88 mm^3^, craniocaudal angle of attack = 36.72°, mediolateral angle of attack = 50.55°; supraorbital: VSF = 43.83 mm^3^, craniocaudal angle of attack = 20.86°, mediolateral angle of attack = 30.40°). VSF, volume of surgical freedom; 3D, 3-dimensional. Used with permission from Barrow Neurological Institute, Phoenix, Arizona.

**FIGURE 7:**
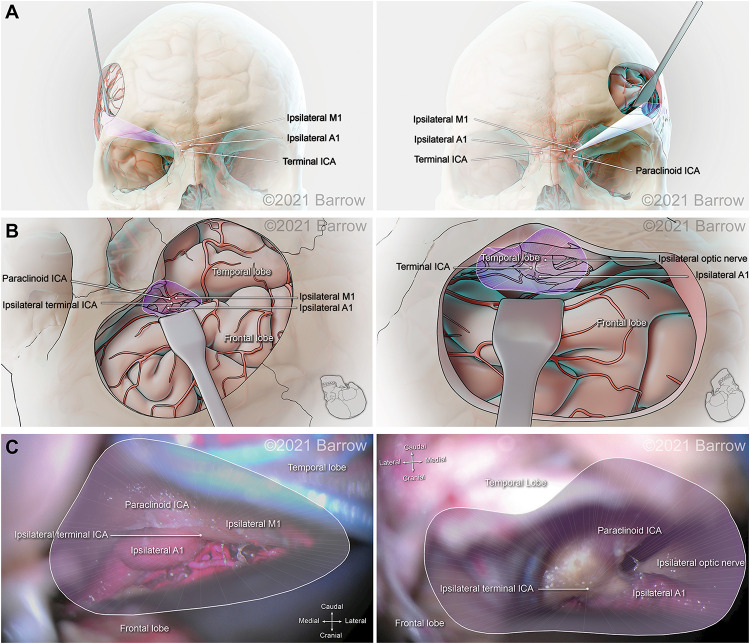
Illustration depicting 3D modeling of the surgical corridor to the terminal internal carotid artery (ICA) from a pterional approach (left) and a supraorbital approach (right). **(A)** The anterior of the surgical corridor. **(B)** An illustration of the surgical anatomy as visualized specific to the surgical corridor model. **(C)** The surgical view of the cadaveric anatomy, which is in continuity with the surgical corridor model parameters (pterional: VSF = 50.69 mm^3^, craniocaudal angle of attack = 22.63°, mediolateral angle of attack = 27.59°; supraorbital: VSF = 31.01 mm^3^, craniocaudal angle of attack = 16.29°, mediolateral angle of attack = 31.62°). ACoA, anterior communicating artery; VSF, volume of surgical freedom; 3D, 3-dimensional. Used with permission from Barrow Neurological Institute, Phoenix, Arizona.

**FIGURE 8:**
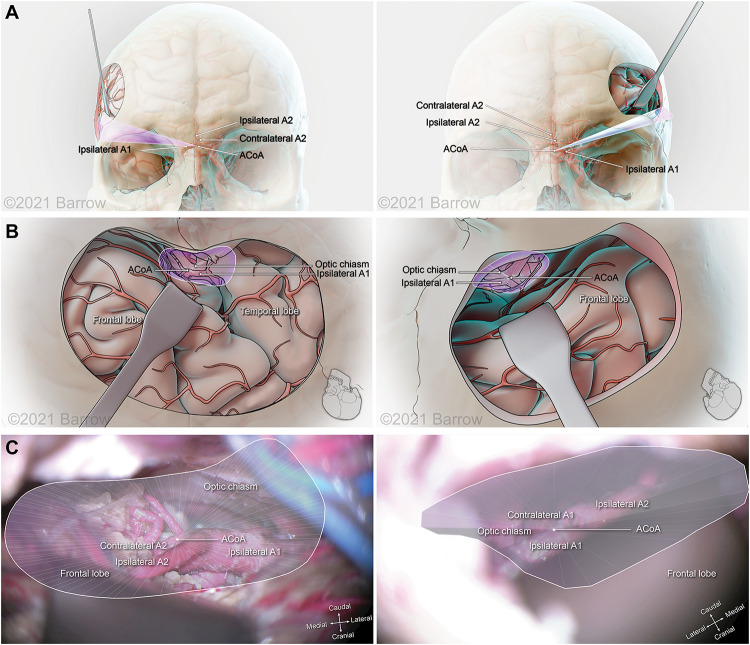
Illustration depicting 3D modeling of the surgical corridor to the anterior communicating artery (ACoA) from a pterional approach (left) and a supraorbital approach (right). **(A)** The anterior of the surgical corridor. **(B)** The surgical anatomy as visualized specific to the surgical corridor model. **(C)** The surgical view of the cadaveric anatomy, which is in continuity with the surgical corridor model parameters (pterional: VSF = 38.34 mm^3^, craniocaudal angle of attack = 12.64°, mediolateral angle of attack = 31.69°; supraorbital: VSF = 15.66 mm^3^, craniocaudal angle of attack = 14.50°, mediolateral angle of attack = 17.44°). ICA, internal carotid artery; VSF, volume of surgical freedom; 3D, 3-dimensional. Used with permission from Barrow Neurological Institute, Phoenix, Arizona.

Figures 6–8 demonstrate the 3D models of the surgical corridors available for deployment of neurosurgery instruments, specific to the pterional and supraorbital approaches and the STSs. This methodology provides an increased body of quantitative and visual information on surgical approach metrics to aid the neurosurgeon in the decision-making process.

### Measurement Inaccuracy

The illustrative data set for Figure 5 was used to compare the calculated area bounded by the original measurement data points to the calculated area bounded by the points after translation onto the best-fit plane. The sample data set comprised measurements for a supraorbital craniotomy surgical corridor to the anterior communicating artery. It represented the area calculated by the previous surgical freedom method (Heron’s Formula) and the area after translation onto the best-fit plane. The calculation of the area bounded by this sample set of data point measurements was 1,489 mm^2^, and the calculation of the area bounded by the data points after translation onto the best-fit plane for the data set was 1,418 mm^2^. To illustrate the effect of probe length on the calculated volume, we used this same data set to calculate the volume from the best-fit plane of the measured data points to the apex. A cone height of 183.6 mm gave a volume of 119,386 mm^3^. The points representing data of the same corridor as measured were then translated to a plane 5 mm farther away from the apex. This 5-mm increase in the height of the same cone shape, to 188.6 mm, gave a cone volume of 129,448 mm^3^.

**FIGURE 9:**
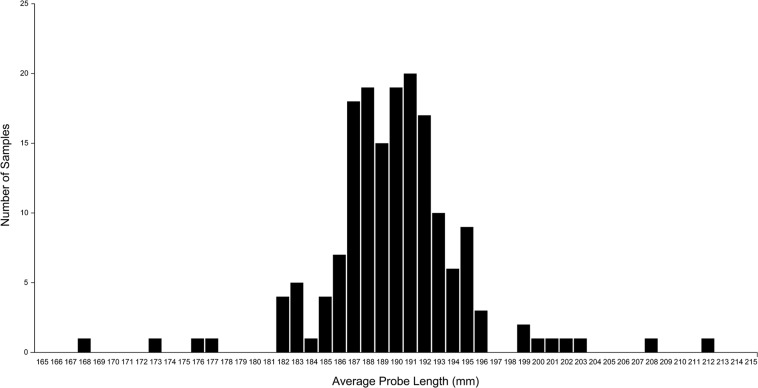
A plot of the average probe length of each set of measurement data. Probe length was calculated as the distance between the coordinates of each extrema point and the apex point. The plot shows considerable variation in the calculated probe length, although the same fixed-length probe was used in all measurements. This variation is caused by measurement error that can be mitigated by using the normalized volume of surgical freedom metric. Used with permission from Barrow Neurological Institute, Phoenix, Arizona.

Figure 9 shows the frequency distribution of the probe lengths of the 8 data points for the 174 sets of measurements. The mean (SD) probe length was 190.4 (5.0) mm, with a minimum of 168.3 mm and a maximum of 212.0 mm.

## Discussion

### Surgical Application Advantages of the VSF

The VSF compensates for multiple defects in the established method of producing a quantitative result to assess surgical instrument freedom. The process incorporates various spatial, anatomical, and technical components pertinent for the accurate and insightful analysis of a surgical corridor. This quantitative approach resolves issues in assessing this surgically imperative parameter that are cause by multiple planes, irregularly shaped access corridors, procedural variability, and mathematical inaccuracies.

This methodology comprises the various embodiments of a system and an associated mathematical method to determine a 3D VSF before operating on neurosurgical structures. The system characterizes, assesses, and models a 3D volumetric measurement of the maneuverability of a surgical instrument within a surgical corridor with respect to STS access, thereby providing new insight into the accessibility of an intracranial structure via a specific approach. It is explicitly advantageous to medical specialties, such as neurosurgery, that deal with microanatomical structure and require competency in microsurgical techniques. Dealing with small structures that have definitive targets is inherent in the microsurgery of STSs that lie at a depth from the surgical entry point.

Neurosurgeons try to maximize the potential physiologic space at their disposal to minimize circumferential damage. The VSF is a metric that enables a more accurate assessment of the freedom of this physiologic space in conjunction with specific surgical maneuvers. Anatomically, this concept and the basis behind neurosurgical corridor modeling can be characterized by examining the pathologic processes encountered by the neurosurgeon. For example, vascular aneurysms are abnormal protrusions or weaknesses in the wall of a blood vessel. Obliteration of these weaknesses is imperative to prevent intracranial hemorrhage and potential morbidity and mortality. Minimizing the degree of brain retraction is an important factor in decision-making when selecting a surgical approach. With the VSF, the degree of retraction can be quantitatively measured with respect to each approach and each STS. Another example is the midline tumor, such as the pituitary lesion, which often produces complex surgical conditions because of the need to cross neurovascular structures and overcome difficult angles of attack produced by the anatomy. As with vascular lesions, the VSF can provide a more accurate quantitative assessment of midline tumors and better anatomical visualization of the different surgical corridors to reach this region of interest. Finally, a third example is the abnormal lesion of the nerves, such as an acoustic neuroma growing at the deep apex of the cerebellopontine angle of the skull base, the trajectory of which naturally follows the conical structure of our model when the potential physiologic space is created by brain retraction and the release of cisternal cerebrospinal fluid.

Given that neurovascular structures tend to traverse the fossae floors along their pathway, the use of this surgical corridor modeling method is particularly relevant in surgical interventions of the skull base, where the STSs are usually bounded inferiorly by the skull base. Skull base surgery usually entails both anatomical and technical obstacles: deep STSs, multiple eloquent structure boundaries, dural tethering, and skull base canal insertion. These influential factors substantially limit maneuverability, which is extremely precious. The VSF is a valuable tool for evaluating surgical corridors that allow only restricted movement and therefore is a particularly useful metric for the assessment of skull base surgical approaches.

This methodology generates the first spatially accurate model of an irregular surgical corridor that also considers actual anatomical boundaries. Furthermore, it allows the influence of the different visualization techniques used in surgical intervention to be examined. For example, the operating microscope, although limited in image expanse and illuminating capabilities, does not function within the approach corridor. In contrast, the endoscope is used within the surgical corridor, where, as an extra instrument or a space-occupying entity, it will impede the freedom of other instrumentation. Experimental scrutiny of this variable has not been completed, although it is common knowledge among endoscopic surgical specialists who have proposed multiportal endoscopic approaches to combat instrument crowding or “swording” (Dallan et al., 2015; Lim et al., 2020). The VSF not only provides a numerical representation of the effect of the endoscope on instrument freedom but also creates a 3D representation of this influence. Notably, the endoscope is dynamic and can be moved out of the trajectory of attack as dictated by the operating surgeon, but the numeric change in the VSF caused by its presence remains valid. Given the dynamic nature of the endoscope, mapping its optimal position in the context of different STSs and angles of attack could pose a useful predictive model for improving the efficiency of surgical movement and minimizing exposure.

The system and the associated method provide the surgeon with a volumetric metric to determine the appropriateness and utility of a surgical approach to access a specific pathology. It would, therefore, potentially allow the neurosurgeon to select approaches and define a safe access corridor for guidance during both the planning and the conduct of surgery. This metric can also elucidate the appropriateness of surgically attainable targets specific to an approach. Like all attempts at neuroanatomical quantitation, the VSF functions as a quantifiable metric for assessing the likelihood of surgical risk and injury to anatomical STSs, but it achieves this with substantially superior accuracy and volumetric spatial computation.

### Procedural Superiority of the VSF

The VSF methodology includes the calculation of the area bounded by the surgical corridor extrema coordinate points. Perpendicular to the central axis of the surgical corridor, this area is bounded by the surgical corridor extrema points that are measured.

Previous methods also measured the bounded area and used this area calculation as the metric of surgical freedom. These methods used Heron’s formula to calculate the area, which involved subdividing the bounded area into triangles, with the measured points as the vertices of the triangles. With the coordinates of each vertex known, the lengths of the three sides of each triangle were calculated using Heron’s formula, and the areas were summed to give the total bounded area.

**FIGURE 10:**
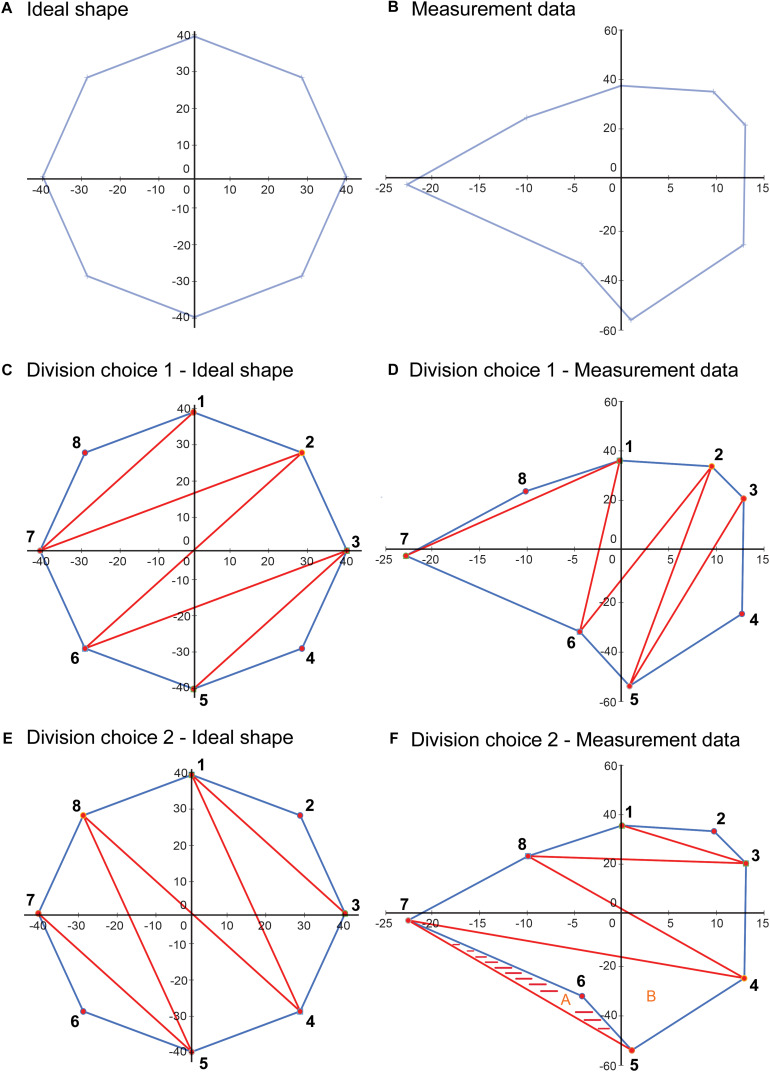
Illustration depicting a limitation of using Heron’s formula to calculate the area of an irregular closed shape. **(A)** An idealized shape made up of eight data points. **(B)** A shape plotted from a sample measurement, also with eight data points. **(C)** An example of a choice of division of the area into component triangles for application of Heron’s formula. **(D)** This division choice applied to the sample data. In this case, using Heron’s formula on each of the triangles results in a correct calculation of the total area. **(E)** Another example of a choice of division of the area into component triangles. **(F)** This division choice applied to the sample data. In this case, using Heron’s formula on each of the triangles results in an overestimation of the bounded area, as the shaded area is included twice in the calculated area, as part of triangles A (5-6-7) and B (4-5-7), although it is outside the area bounded by the data points. Used with permission from Barrow Neurological Institute, Phoenix, Arizona.

In our proposed VSF methodology, the bounded area is used in calculating the VSF because the base of the cone shape is formed by the bounded area and the area value is used to calculate the volume of the cone shape. The VSF methodology uses a different method—the shoelace formula—to calculate the area. The shoelace formula was used instead of Heron’s formula because it can calculate the area of irregular shapes more accurately. Heron’s formula inherently requires choosing how to divide the area into triangles, which is important, as illustrated in Figure 10. This figure illustrates two methods of dividing an ideal shape and a shape determined from measurement data into triangles. The first choice of division of the shape results in an accurate calculation of the bounded area of each shape. However, the second choice of division, while calculating the area of the ideal shape correctly, overestimates the area of the shape from the measurement data and results in an area outside the bounds of the shape being included in the calculation.

A method that requires a choice, or the validation of a choice, for each set of data is not conducive to creating a calculator that will give repeatable and accurate results because each calculation requires user input to review and determine how the shape will be divided into triangles. To avoid this dilemma, we selected the shoelace formula to use in calculating the area for the VSF methodology. This formula uses coordinate data of the vertices of a closed, irregular shape to calculate the enclosed area. This method accounts for irregular shapes and does not overestimate the area, as the Heron’s formula method does. Because the VSF method includes the identification of a normalized-height plane for the base of the cone, and the measured points are translated onto this plane, the points can be converted into a 2D coordinate system on the normalized-height plane, and the 2D shoelace formula can be used. Converting the 3D coordinates to 2D coordinates has the advantage of allowing the creation of 2D plots of the cone base that can be used to verify the measurement data.

Translating the original measurement points onto a plane and calculating the area using the translated points also increases the accuracy of the calculated area over a calculation using the original 3D measurement points. The area of interest is the area of the surgical corridor perpendicular to the central axis of the surgical corridor, which is the truest representation of the accessibility of the surgical approach. Because the best-fit plane is perpendicular to the central axis of the surgical corridor, the area bounded by the translated points is perpendicular to the axis of the surgical corridor and thus represents a true cross-section of the surgical corridor. As noted previously, the 3D coordinate data that are measured are not in a single plane because of inaccuracies in measurement, meaning that the triangles formed for measuring the bounded area using Heron’s formula are not perpendicular to the central axis of the surgical corridor. As a result, calculating the total area by summing the areas of the triangles would be an overestimation. The sample data set of Figure 5 illustrates this as the area calculated using Heron’s formula on the original measurement data points (1,489 mm^2^) was 5% higher than the perpendicular area measured from the data points after translation onto the best-fit plane (1,418 mm^2^).

### Benefits of Using Normalized Height

Although the concept of volumetric quantification of surgical freedom is novel, we further refined the concept to a normalized volumetric quantification of surgical freedom to compensate for several inaccuracies inherent in the measurement process. The measurement process involves measuring the coordinate data points for the extrema of the surgical corridor at the end of a surgical instrument of fixed length (Figure 4). Doing so resulted in the coordinate data being measured outside the cadaveric specimen, such that the area being measured was not a measurement of the surgical entrance but rather an abstract measurement of surgical freedom that could only be compared to other measurements that used a surgical instrument of equal length. Measuring with a shorter probe would result in a smaller measurement of surgical freedom for the same surgical corridor because of the conical shape of the surgical corridor, whereas measuring with a longer probe would result in a larger measurement of surgical freedom because the length of the probe defines the vertical height of the cone shape from base to apex.

The depth of the structure from the surgical entry point is of secondary importance to the degree of freedom of the instruments within the surgical corridor, which is why this method of measurement can be used at the end of a fixed probe. With the use of this method, surgical corridors and their quantitative measurements with respect to STSs can be directly compared only if the probe length is exactly equal across studies. This limitation raises questions about the scientific validity of these studies and restricts replication of results and large-scale analysis of anatomical surgical corridor data. The limitation is equally applicable to a volumetric measurement of surgical freedom; for the same surgical corridor, a shorter probe results in a smaller volume measurement, and a longer probe results in a larger volume measurement. Measuring the volume of the surgical corridor up to a fixed distance from the apex (a normalized height) can mitigate this issue. The measurement data from any length of probe can be used to calculate the VSF at a fixed distance from the apex, so the normalized VSF calculation allows the direct comparison of VSF data from any measurement that uses this method.

This decoupling of cone height from probe length also improves the accuracy of the VSF measurement. In our experimental data, human error in collecting coordinate data led to variation in the distance between the apex point and each of the extrema data points of the surgical corridor. This inaccuracy in the data existed in all three dimensions, and there is little that can be done in processing the data to improve the accuracy in the two dimensions on the plane of the base of the cone. However, the normalized VSF measurement, by defining a fixed distance from the apex to the base of the cone, can reduce the inaccuracy in the third dimension along the axis of the cone. The inaccuracy in this dimension has a large effect on resultant calculations. The SD of the average probe length data for all measurement data sets was identified as 5 mm from the plot (Figure 9). The effect of this variation on the calculated volume can be seen in our sample data set analysis. In this case, the height of the cone shape increased from 183.6 to 188.6 mm, for an increase of 5 mm (2.7%). The resultant calculation of the cone volume increased by 8.4%. This outcome demonstrates how errors in probe length measurements can be exaggerated in the calculated surgical freedom metric, and thus may translate to important surgical implications. The use of a normalized cone height eliminates this variation in the calculated volume and leads to a more consistent result that can be compared to other normalized VSF data with a much higher degree of confidence.

### 3D Modeling of the Operative Corridor

Visualization is a critical skill. From the surgeon’s perspective, being able to orient and envision the structures and any restrictions and obstacles in the surgical corridor is imperative to selecting the most appropriate approach and planning specifically for the selected surgical strategy. As previously noted, neuroanatomy has been extensively analyzed and neurosurgeons have a well-established knowledge base about anatomical sites and the sizes and arrangements of specialized regions. Less explicitly defined are the physiologic and surgical corridors created by operators, the architecture of these potential or created spaces, and how the circumferential anatomy affects these crucial aspects in surgical decision-making.

The novel design of the VSF results in an improved concept constructing a configuration that embodies the actual geometry of the surgical approach corridor, which is illustrated by our models. When incorporated with the clinical, anatomical, and surgical application, this volumetric model yields better assessment and prediction of the ability to manipulate surgical instruments, while providing spatially and anatomically accurate representation that can aid the surgeon in decision-making. These images accentuate the importance of not only anatomical considerations but also the critical principles of microsurgery: technique, instrument maneuverability, and the predicted primary instrument axis. The VSF methodology provides an anatomically and spatially accurate 3D depiction illustrating all these key surgical ideals, which proves its substantial clinical applicability.

### Limitations

Ideally, this quantitative analysis and modeling should be conducted *in vivo* in human patients rather than in cadaveric specimens because the brain parenchyma can harden in cadavers with fixed tissue, resulting in decreased surgical maneuverability. However, cadaveric brain tissue is the best model available; although it may not be exactly representative, the quantitative results are proportionate. Although the surgical corridor may be larger *in vivo*, the anatomical parameters are the same, and the 3D models of the surgical corridor are therefore still reflective of real-time surgical views.

Ideally, the VSF measurements should be made in relation to pathologic processes, such as a vascular weakness like an aneurysm or an intracranial mass lesion, which was not possible in the current study. We therefore could not take into account the potential mass effect influence of intracranial pathology on surrounding brain parenchyma and structures, nor could we predict the pathologic decrease in intracranial potential space. Again, this limitation is inherent in all cadaveric modeling because the accessible anatomy is generally physiologically normal. The reproducible STSs are reasonable representations of delicate neurovascular components of high priority to the neurosurgeon who must select the optimal approach on the basis of quantitative metrics that are critical to the decision-making process. What can be extrapolated from our analysis is the predictable numeric value and anatomical shape of surgical access corridors used to reach the pathologic target. In addition, the VSF quantitatively and visually allows for the comparison of approaches, and it ultimately provides increased multifaceted information for surgical decision-making that is comparable to other available metrics.

**FIGURE 11:**
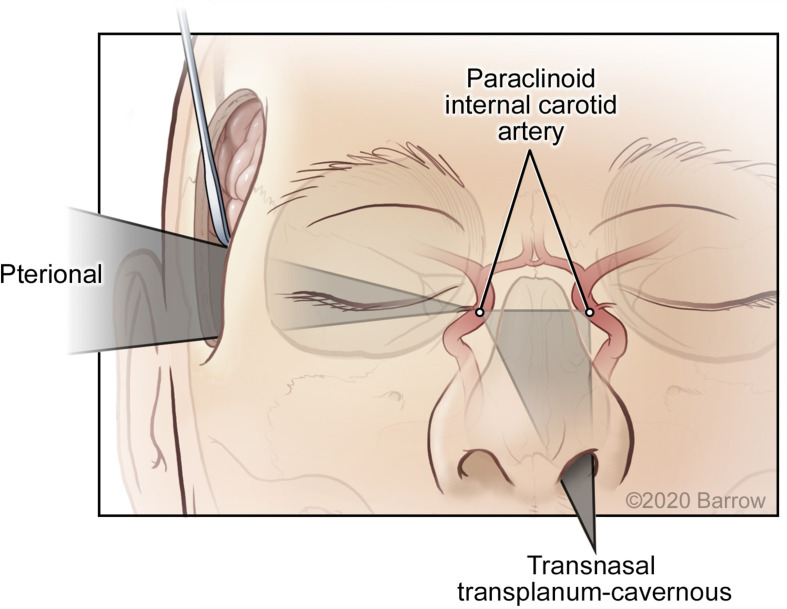
Surgical maneuverability of an instrument using a transcranial pterional approach compared with a transnasal transplanum-cavernous approach to the paraclinoid internal carotid artery. In the transcranial approach, the surgical corridor progresses from a superficial region of large maneuverability to a small deep apex, whereas in the transnasal approach, a significant amount of deep volume is created by traversing the ethmoid and sphenoid sinuses. Used with permission from Barrow Neurological Institute, Phoenix, Arizona.

The VSF methodology is based on the assumption that the surgical corridor traverses from a region of large freedom and maneuverability to an apex or an STS. This assumption produces the cone shape that supports the mathematical and modeling structure of this system. This is the accepted surgical trajectory of transcranial surgical interventions and potentially that of other surgical interventions, where instruments proceed from areas of large surgical freedom to small, confined regions necessitating microsurgical technique. However, this model is not applicable for comparison with all approaches. The caveat of this quantitative and visual estimator is the assumption of the surgical apex; the corridor ends at the point represented by the STS. For quantitatively and spatially comparable results, the comparison of different surgical approaches is possible only when both approaches abide by this assumption. For example, the comparison of a transcranial pterional approach and an endonasal transplanum-cavernous approach to the paraclinoid internal carotid artery is not an equivalent assessment, because an endonasal approach creates a large amount of deep surgical exposure, and its parameters do not converge to an apex as in a transcranial approach (Figure 11). Conversely, it is acceptable to quantify and model specific to an STS if both approaches have the same surgical boundaries (i.e., both use a deep transsphenoidal approach) and if an assumption has been established that the STS represents the conical apex. For example, a comparison of a transnasal approach and a transmaxillary approach is possible because both produce the same deep surgical exposure, although they are restricted more superficially by different anatomical structures at the surgical entrance. In this scenario, the VSF is a useful metric and a helpful surgical corridor modeling tool for visualizing these restrictions.

### Future Directions

This report describes in detail an improved and more representational mathematical and modeling methodology for quantitatively assessing surgical freedom. Our rationale was to produce a robust, multifaceted tool that neurosurgeons can use to estimate the benefits and disadvantages of different surgical approaches specific to various STSs.

Although we have illustrated quantitatively and visually how the various constituents of this novel design are superior to the previous method, comparative analysis has yet to be completed. Our next step in proving the scientific, mathematical, logistical, and applicable advantages of the VSF will be to complete cadaveric quantitation of the same surgical approaches specific to various STSs and to analyze the results. Specifically, three areas should be examined in detail when comparing this VSF methodology with previous methodologies: (1) the increase in accuracy of the measured area when measuring the area on a single plane versus on points in 3D space because of the measurement of only the perpendicular area; (2) the reduction in the variation in results because of the calculated probe length using the normalized unit of the VSF and how variations in probe length affect the calculated area or the VSF metric; and (3) further analysis of the limitations of Heron’s formula, specifically regarding the choice of division of the bounded area of irregular shapes.

Our interdisciplinary research group also intends to replicate this experimental methodology with multiple neurosurgical approaches. We will then quantitatively analyze the benefits and disadvantages of different operative corridors to specific STSs, which will ultimately increase the body of reproducible, standardized, and comparable surgical freedom data and promote optimal surgical techniques and practices.

In regard to comparing the VSF with the previously used method, we predict that the quantitative results will correlate and will be approximately proportionate. It is important to highlight that the merits of the VSF are a result of two key features: the increased accuracy of this multifaceted biomedically orientated mathematical methodology and the ability to produce anatomically and spatially accurate 3D models. These two components have coalesced to produce an effective translational tool that combines anatomical, clinical, and surgically pertinent principles for improved operative decision-making.

Although our report details the use of this methodology for neurosurgical operative corridors, this system can likely be used in its current form to quantitatively analyze and spatially visualize the surgical approaches of different surgical specialties. Doing so would require that they abide by the structural parameters of this measurement process: that the instrument freedom of the surgeon traverses from a region of larger maneuverability at the surgical entrance to a target apex of minimal maneuverability. As denoted when referencing skull base surgical concepts, it is in fixed domains of minimal freedom that knowledge and insight about the movement capabilities of an instrument are most important. Quantitation of spinal surgical corridors and specific STSs is certainly feasible using this methodology, and it is worthy of further investigation.

Our method for determining the VSF provides the surgeon with a diverse metric and a useful tool for improved surgical preoperative planning and decision-making. Current neurosurgical navigational systems plan surgical routes along a direct trajectory based on a linear display. These navigational approaches portray the trajectory line to the STSs in different views (e.g., axial, coronal, sagittal, and probe view), which is not always the optimal approach and does not incorporate any criteria relevant to surgical corridor analysis. These planning navigation systems also do not incorporate any modeling, which would elucidate or illustrate the degree of surgical freedom. By compiling a substantial body of data, we hope to develop standardized reproducible surgical approach principles specific to operative corridors and STSs and thereby establish predictive surgical theory. Consequently, the integration of the VSF into intraoperative planning, as well as into surgical navigation software and systems, could prove to be a powerful tool for improving surgical decision-making and techniques, while ultimately minimizing surgical morbidity and mortality.

## Conclusion

The VSF is a superior method of quantitative anatomical measurement. This innovative concept can be used to develop an actual geometric model of a surgical corridor that yields better assessment and prediction of the ability to manipulate surgical instruments. The VSF accounts for multiple inaccuracies in the practical and mathematical method of assessment, and it also enables the production of 3D models. For this reason, the VSF is a preferable and clinically applicable standard for the assessment of surgical freedom. This quantitative measurement can establish surgically attainable targets for specific approaches and also assess the suitability of a specific surgical approach compared to alternative operative options, thereby acting as a pivotal tool in the decision-making armamentarium of the neurosurgeon.

## Data Availability Statement

The raw data supporting the conclusions of this article will be made available by the authors, without undue reservation.

## Author Contributions

LMH: conception, design, data collection, data analysis, manuscript write-up, review, and revisions. DN: conception, design, data Analysis, manuscript write-up, review, and revisions. MCP: supervision, review, and revisions. All authors contributed to the article and approved the submitted version.

## Conflict of Interest

The authors declare that the research was conducted in the absence of any commercial or financial relationships that could be construed as a potential conflict of interest.

## Abbreviations

ACoA: anterior communicating artery
ICA: internal carotid artery
SD: standard deviation
STS: surgical target structure
VSF: volume of surgical freedom
2D: 2-dimensional
3D: 3-dimensional.
